# Bilateral nephromegaly due to direct leukemic cell invasion in the initial and relapse phases of T-cell acute lymphoblastic leukaemia: A case report

**DOI:** 10.1097/MD.0000000000028391

**Published:** 2021-12-23

**Authors:** Yoshifuru Tamura, Ritsu Sumiyoshi, Tadashi Yamamoto, Yuto Hayama, Yoshihide Fujigaki, Shigeru Shibata, Yuko Sasajima, Haruko Tashiro

**Affiliations:** a Division of Nephrology, Department of Internal Medicine, Teikyo University School of Medicine, 2-11-1 Kaga, Itabashi-ku, Tokyo, Japan; b Department of Hematology/Oncology, Teikyo University School of Medicine, 2-11-1 Kaga, Itabashi-ku, Tokyo, Japan; c Department of Pathology, Teikyo University Hospital, 2-11-1 Kaga, Itabashi-Ku, Tokyo, Japan.

**Keywords:** acute kidney injury, bilateral nephromegaly, positron emission tomography, renal infiltration, T-cell acute lymphoblastic leukaemia

## Abstract

**Rationale:**

T-cell acute lymphoblastic leukemia is a relatively uncommon disorder in adults. Kidneys are not frequently invaded by leukemic cells, and patients with adult ALL showing nephromegaly as an initial presentation are rare.

**Patient concerns:**

A 54-year-old man was referred to our institution for mild anemia and thrombocytopenia. Laboratory tests showed bicytopenia with abnormal lymphoid cells in the peripheral blood and mild renal dysfunction.

**Diagnosis:**

Ultrasonography and computed tomography (CT) revealed bilateral enlargement of the kidneys. [18F]-fluorodeoxyglucose positron emission tomography/CT demonstrated a strong increase in metabolic uptake in the bilateral kidneys. A kidney biopsy revealed a leukemia invasion into the parenchyma. Based on the lymphocytic repertoire, the patient's condition was diagnosed as T-cell acute lymphoblastic leukaemia.

**Interventions:**

The patient received hyper-cyclophosphamide, vincristine, adriamycin, and dexamethasone and high-dose methotrexate and cytarabine as induction chemotherapy. After his leukemia relapsed, he received nelarabine as a second induction therapy and underwent haploidentical peripheral blood stem cell transplantation.

**Outcomes:**

Complete remission (CR) was achieved after chemotherapy. Chemotherapy also improved renal function associated with the normalization of bilateral nephromegaly. Repeated [18F]-fluorodeoxyglucose - positron emission tomography/CT posttreatment showedregression of metabolic uptake in the bilateral kidneys. The patient underwent cord blood transplantation at the first CR, but his leukemia relapsed 9 months later. At relapse, bilateral nephromegaly reappeared. Then, the second induction therapy induced CR for at least 10 months after induction therapy.

**Lessons:**

Although rare, ALL in the initial and relapsed phases can be associated with bilateral nephromegaly and renal impairment due to the invasion of leukemic cells into the parenchyma with or without abnormal leukemic cells in circulation. Leukemia is an important differential diagnosis of renal impairment with bilateral nephromegaly.

## 1 Introduction

T-cell acute lymphoblastic leukemia (T-ALL) is an immature lymphoid tumor that preferentially localizes in the bone marrow, lymphoid organs, and central nervous system, particularly in the mediastinal lymph nodes. Kidneys are not common organs in which leukemic cells directly infiltrate at the time of diagnosis. T-ALL is a relatively uncommon disorder in adults, with an incidence of 0.13 cases/100,000 population in the United States.^[1]^ Although there are several reports on pediatric ALL patients who developed nephromegaly at diagnosis, adult T-ALL cases with nephromegaly as an initial presentation are extremely rare.^[2,3]^ Additionally, acute kidney injury (AKI) due to direct leukemic cell invasion into the kidney in T-ALL is uncommon at the time of leukemia diagnosis.^[4]^ Herein, we present the case of an adult T-ALL patient who presented with bilateral nephromegaly and AKI at leukemia diagnosis.

## 2 Case report

**Figure 1:**
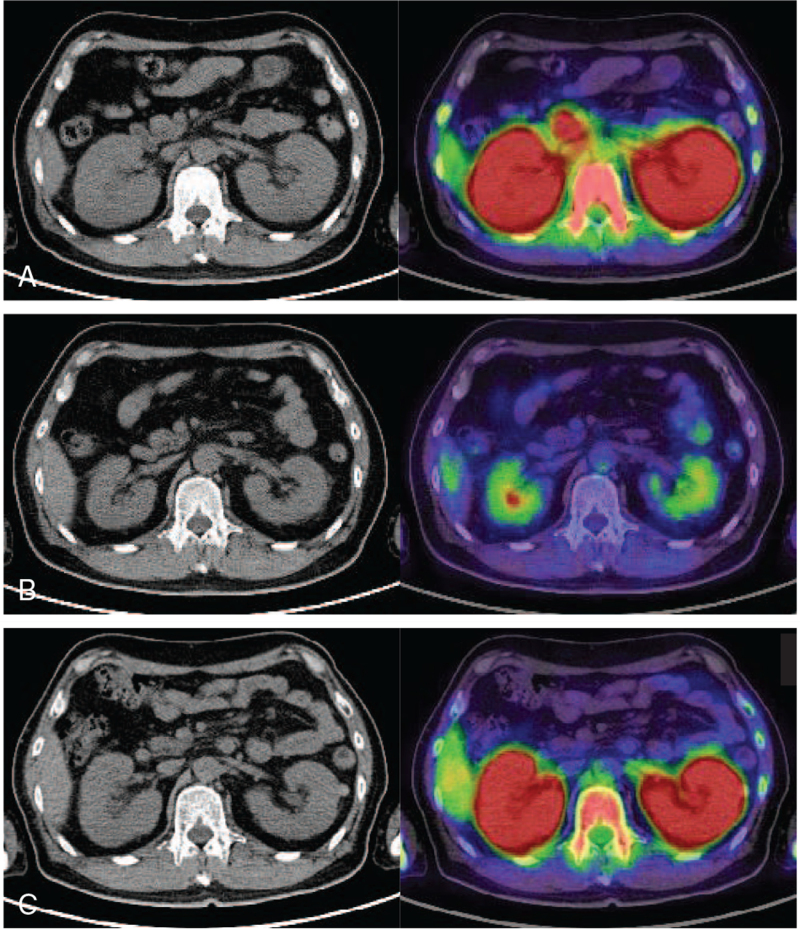
Pre-and posttreatment 18-fluorodeoxyglucose (^18^FDG) positron emission tomography (PET)-computed tomography (CT) of the abdomen (A) ^18^FDG PET/CT at diagnosis showing ^18^FDG-strong uptake in bilateral enlarged kidneys. (B) ^18^FDG PET/CT posttreatment showing no abnormal ^18^FDG uptake in the bilateral kidneys. (C) ^18^FDG PET/CT at relapse showing ^18^FDG-strong reuptake in bilateral enlarged kidneys.

A 54-year-old man with a history of hypertension was referred to our institution with dizziness, mild anemia, and thrombocytopenia. His general condition was good, and his blood pressure was 120/74 mmHg. Physical examination findings were unremarkable, with no lymphadenopathy or hepatosplenomegaly. Laboratory data showed the following values: white blood cells, 4,600/μL with 6% of abnormal cells which were negative for myeloperoxidase; hemoglobin, 8.1 g/dL; platelets, 88,000/μL; lactate dehydrogenase, 211 U/L; serum creatinine, 1.59 mg/dL; estimated glomerular filtration rate, 37.2 mL/min/1.73 m^2^. Urinary chemistry revealed the following findings: urinary protein-creatinine ratio, 1.19 g/g creatinine; and urinary red-blood-cell, 0-1/ high-power field; Nacetyl-β-D-glucosaminidase, 4.5 U/L; β 2-microglobulin, 5,000 μg/L; and alpha 1-microglobulin, 151 mg/L, indicating advanced tubulointerstitial injuries. The immunological findings were as follows: C-reactive protein, 0.92 mg/dL; IgG, 1,520 mg/dL; IgA, 231 mg/dL; IgM, 49 mg/dL; IgE, 125 IU/mL (normal < 100); hepatitis B and C serology, normal; human immunodeficiency virus antibody test, negative; anti-nuclear antibody, negative; anti-DNA antibody, negative; myeloperoxidase and proteinase 3 ANCAs, negative; and anti-glomerular basement membrane antibody. Although he underwent an annual health check-up, the kidney dysfunction had never been identified. From the myeloperoxidase-negative abnormal cells in the peripheral blood, we suspected that the underlying disease was lymphoma with bone marrow infiltration. Abdominal ultrasonography and computed tomography (CT) of the chest, abdomen, and pelvis showed enlargement of the bilateral kidneys (12 cm on the right and 13 cm on the left) with no other significant findings, including lymphadenopathy. Further diagnostic work-up of [18F]-fluorodeoxyglucose (^ 18^FDG)-PET/CT demonstrated a strong increase in metabolic uptake in the bilateral kidneys (Fig. 1A). Since a bone marrow aspiration showed dry tap, we performed a bone marrow biopsy that later revealed T-ALL. Although renal involvement of acute lymphoma or leukemia was strongly suspected in our patient, the exact cause of non-oliguric AKI with isolated proteinuria is unknown. A percutaneous kidney biopsy was safely performed with an appropriate blood transfusion for thrombocytopenia.

**Figure 2:**
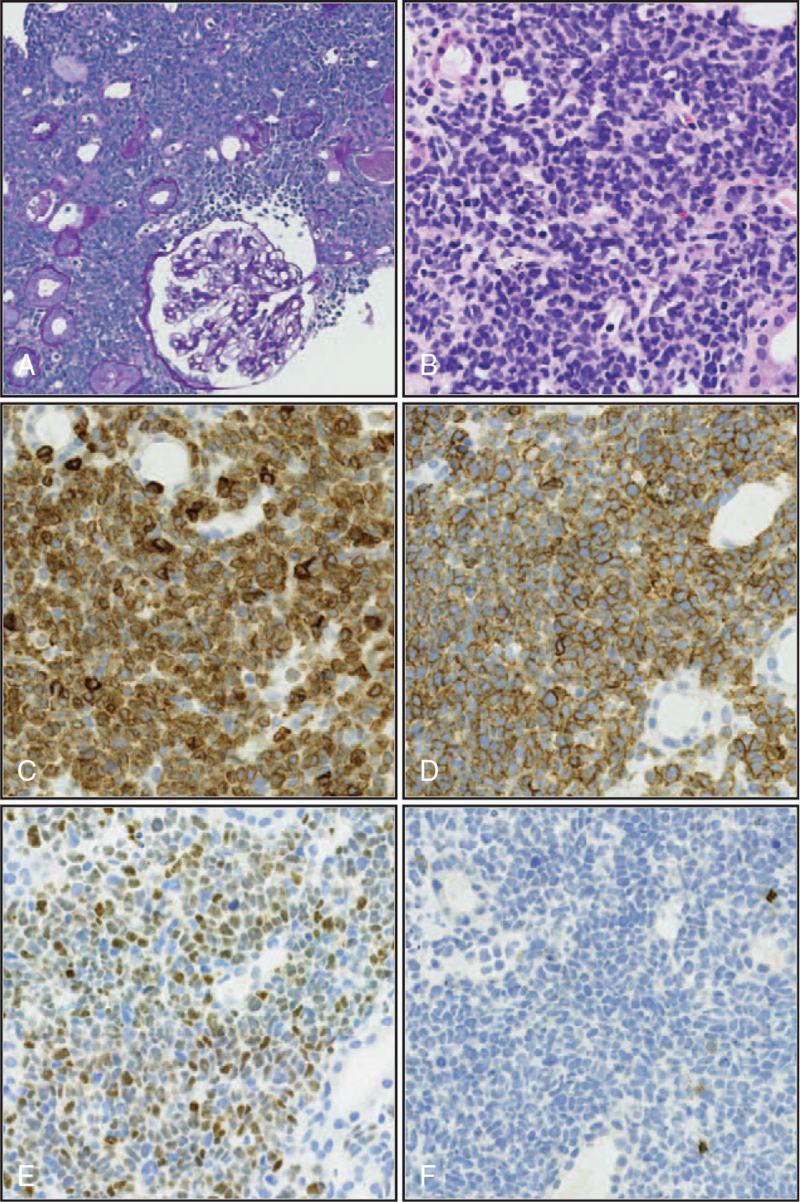
(A) Periodic acid-Schiff staining (20×) showing an extensive infiltrate obliterating the kidney parenchyma; (B) Hematoxylin and eosin staining (40×) showing tumor cell morphology; (C) Immunohistochemistry (IHC) showing (40×) CD3-positive tumor cell; (D) IHC showing (40×) CD5-positive tumor cell; (E) IHC showing (40×) TdT-positive cells; (F) IHC showing (40×) CD20-negative tumor cell. TdT = terminal deoxynucleotidyl transferase.

A kidney biopsy showed six glomeruli with minor abnormalities (Fig. 2A). The kidney parenchyma was infiltrated by an extensive mixed lymphoid infiltrate composed of small cells, spindled cells, and a predominance of large lymphoid cells with abnormal nuclei (Fig. 2A, B). Occasionally, mitotic figures were observed (Fig. 2B). There were no signs of tubulitis or arteritis. Immunohistochemistry showed that these lymphoid cells were positive for CD3, CD5, and terminal deoxynucleotidyl transferase, with negative staining for CD20 (Fig. 2B-F). Additionally, Ki-67 was highly expressed in these blasts (not shown). Immunofluorescence and electron microscopy revealed no evidence of immune deposits. These findings confirmed tubulointerstitial T-ALL.

After the diagnosis of T-ALL, the patient received hyper-cyclophosphamide, vincristine, adriamycin, and dexamethasone as induction chemotherapy. He did not develop tumor lysis syndrome or renal failure, and his creatinine level promptly decreased to normal levels after chemotherapy. He also received high-dose methotrexate and cytarabine, which induced complete remission (CR). After 3 courses of chemotherapy, positron emission tomography (PET)/CT showed normal kidney size without abnormal ^18^FDG uptake (Fig. 1B). Although he underwent cord blood transplantation at the first CR, his leukemia relapsed 9 months later. At relapse, in addition to bone marrow relapse, bilateral nephromegaly reappeared (Fig. 1C). Nelarabine induced a second CR, and he underwent peripheral blood stem cell transplantation from his daughter as a second stem cell transplantation. His leukemia had been in CR for at least 10 months after the second transplantation.

## 3 Discussion and conclusion

The present case showed a rare occurrence of AKI associated with bilateral nephromegaly secondary to T-ALL infiltration into the bilateral tubulointerstitium at the time of T-ALL diagnosis. Renal infiltration of ALL is usually observed in the late stages of the disease.^[5]^ Only a few pediatric cases have been reported to show bilateral renal infiltration, causing nephromegaly at diagnosis.^[6,7]^ To the best of our knowledge, there are only three reported adult cases of bilateral nephromegaly due to leukemia infiltration at diagnosis.^[2,3,8]^ Additionally, two adult ALL cases showed bilateral nephromegaly at the time of relapse after stem cell transplantation.^[9,10]^ Interestingly, in these cases, leukemia infiltration into the kidneys was confirmed before bone marrow relapse. Some pediatric cases at diagnosis showed bilateral nephromegaly without abnormal cells in the circulation.^[5]^

In our case, since the patient had renal dysfunction with abnormal cells in circulation and bicytopenia, it was obvious that he had hematological disease. Bilateral nephromegaly can be caused by various conditions, such as diabetes mellitus,^[11]^ acute glomerulonephritis,^[12]^ acute interstitial nephritis,^[13]^ acute tubular necrosis,^[14]^ rapidly progressive glomerulonephritis,^[15]^ and HIV-associated nephropathy,^[16]^ amyloidosis,^[17]^ malignant or benign infiltrative diseases (multiple myeloma, lymphoma, leukemia, and renal metastasis^[18]^), bilateral hydronephrosis, and cystic diseases. Although the incidence is low, renal infiltration by acute leukemia is an important differential diagnosis, even in adult patients. It is not known why leukocytic cells in the present patient have affinity in the kidney. Interestingly, T-ALL relapse was suspected due to the reappearance of AKI and bilateral nephromegaly. In such cases, nephromegaly may be the first indicator of T-ALL relapse.

^18^FDG-PET/CT is used for staging and monitoring therapy response and disease activity.^[19]^ In general, ^18^FDG-PET/CT can detect leukemia throughout the body, although the intensity of uptake varies by.^[20]^ Initially, we thought that his underlying disease was lymphoma; however, PET/CT was performed to determine the disease distribution. Generally, for patients with leukemia, PET/CT is not often performed before induction therapy. This is the first report of ^18^FDG-PET/CT scan of bilateral nephromegaly in adult T-ALL at the time of initial diagnosis. In organs such as the kidney, which frequently show physiological ^18^FDG uptake, it might be difficult to evaluate the involvement of malignant cells using ^18^FDG PET/CT alone.^[21]^ However, in the present case, PET/CT demonstrated a strong increase in the metabolic uptake in the bilateral kidneys. A ^18^FDG PET/CT has been considered one of the most accurate imaging methods for the detection of medullary and extramedullary sites of hematologic malignancies, including the kidneys.

AKI due to lymphomatous infiltration is rare. In a series of 48 patients with aggressive lymphomas with renal involvement, only three patients had AKI.^[22]^ Other causes of renal failure in lymphoma include ureteric obstruction, hypercalcemia, urate nephropathy, sepsis, radiation nephritis, and paraproteinemia.^[23]^ Our patient did not have any of these factors. The mechanisms of renal failure when lymphomatoid cells infiltrate into the tubulointerstitium have not been fully elucidated. However, there are some explanations for this finding. An increase in interstitial pressure due to tumor infiltration, which causes tubular obstruction, compression of peritubular capillaries, and alteration in the tubuloglomerular feedback may cause AKI.^[24]^ In this case, chemotherapy improved renal function and bilateral symmetrical nephromegaly. Furthermore, a second ^18^FDG-PET/CT scan after treatment showed regression of metabolic uptake in the bilateral kidneys (Fig. 1B). Therefore, it is possible that the tubular obstructions by leukemic cells were released by chemotherapy, resulting in the recovery of renal function.

In conclusion, ALL should be suspected in cases of AKI and bilateral enlargement of the kidneys. Modern imaging techniques and renal histology play a supportive role in establishing a diagnosis.

## Author contributions

**Conceptualization:** Yoshifuru Tamura.

**Investigation:** Yoshifuru Tamura, Ritsu Sumiyoshi, Tadashi Yamamoto, Yuto Hayama, Haruko Tashiro.

**Methodology:** Yoshifuru Tamura, Haruko Tashiro.

**Validation:** Haruko Tashiro.

**Visualization:** Yoshifuru Tamura.

**Writing – original draft:** Yoshifuru Tamura, Haruko Tashiro.

**Writing – review & editing:** Yoshifuru Tamura, Yoshihide Fujigaki, Shigeru Shibata, Yuko Sasajima, Haruko Tashiro.
